# Systematic mapping review of applications of extracorporeal shockwave therapy (ESWT) in hand surgery

**DOI:** 10.1016/j.jpra.2025.11.017

**Published:** 2025-11-19

**Authors:** Ryan Faderani, Zhen Yu Wong, Oluwatobi Adegboye, Muholan Kanapathy, Dariush Nikkhah, Afshin Mosahebi

**Affiliations:** a Division of Surgery and Interventional Science, University College London, London, UK; b Department of Plastic Surgery, Morriston Hospital, Swansea, Wales, UK; c Faculty of Biology, Health and Medicine, University of Manchester, Manchester, England, UK; d Department of Plastic Surgery, Royal Free Hospital, London, UK

**Keywords:** Extracorporeal shockwave therapy, Hand surgery, Systematic mapping review, Carpal tunnel Syndrome

## Abstract

**Aim:**

This systematic mapping review aims to consolidate and categorize existing evidence regarding the utilization of extracorporeal shockwave therapy (ESWT) in hand surgery.

**Methods:**

Following the *Preferred Reporting Items for Systematic Reviews and Meta-Analyses (PRISMA)* guideline, a comprehensive search was performed to identify studies assessing ESWT in hand conditions. Eligible studies required a minimum of 3 months’ follow-up. Data extraction adhered to predetermined criteria, and outcomes were classified into clinical assessment, patient-reported outcome measures (PROMs), neurophysiological tests, and miscellaneous domains.

**Results:**

Thirty studies met inclusion criteria, comprising 18 randomized controlled trials (RCTs), 10 case series, and two cohort studies. Evidence was predominantly centered on carpal tunnel syndrome (CTS), with additional studies addressing trigger finger, Dupuytren’s disease, post-carpal tunnel release (post-CTRS) pillar pain and scaphoid wrist nonunions. Most reports demonstrated short-term improvements in clinical and PROM outcomes, with complications infrequent and generally mild. However, treatment protocols varied widely in terms of shockwave type, intensity, and frequency. Observations on dose dependency were inconsistent, and most RCTs carried a moderate to high risk of bias, with all reporting a Fragility Index (FI) of zero.

**Conclusion:**

This review highlights ESWT as a promising adjunct with short-term benefits across select soft tissue conditions of the hand. Nevertheless, its role in long-term management remains uncertain. Standardization of treatment parameters and rigorously designed multicenter RCTs with extended follow-up and functional outcomes are required to establish the durability, safety, and clinical utility of ESWT in hand surgery.

## Introduction

Hand conditions are common presentations to healthcare services globally and if not treated appropriately, they are associated with a significant impact on quality of life and a wider socioeconomic impact.^1^ Extracorporeal shockwave therapy (ESWT) is a relatively novel non-surgical modality that has increasingly been used to manage musculoskeletal conditions, including those of the hand.^2^

ESWT is a non-invasive therapy that delivers acoustic pulses to the target area, it was originally designed for lithotripsy to treat renal stones; however there has been growing trend in its use for musculoskeletal conditions. ESWT has shown several potential benefits when managing musculoskeletal conditions, such as alleviating pain and improving function, leading to a notable popularization over the past few years, especially within orthopedics, physiotherapy, and sports medicine^3^^,^^4^ supported by various biological studies. It operates through different mechanisms on bone, tendon, and skin as well as pain control.^5^, ^6^, ^7^, ^8^ For one-related conditions such as scaphoid wrist nonunions and Kienböck’s disease, ESWT appears to promote healing in fractures and tendon-to-bone healing, potentially through mechanotransduction pathways, though evidence for shockwave-induced micro-fractures is limited.^9^ For tendon pathologies, ESWT may be beneficial in conditions like trigger finger and de Quervain’s tenosynovitis, where its anti-inflammatory effects contribute to reducing fibrosis, improving lubricin production for tendon gliding, and accelerating extracellular matrix biosynthesis.^10^^,^^11^ This effect extends to Dupuytren’s disease, where ESWT could potentially modulate fibroblast activity and reduce contracture progression, although further studies are needed. In post-carpal tunnel release (CTRS) pillar pain, ESWT's ability to decrease nerve fiber excitability and modulate pain pathways suggests a role in post-surgical pain management, potentially reducing reliance on prolonged conservative measures.^12^ Similarly, in hypertrophic hand scars, ESWT has been shown to enhance neocollagenesis and neoangiogenesis, improving collagen organization and skin elasticity, making it a potential adjunct in scar remodeling.

ESWT’s recent growth is attributed to its non-invasive nature and technological advancements that make devices more practical. Moreover, contemporary ESWT studies on musculoskeletal conditions have displayed positive patient outcomes and tolerable side effect profiles.^13^ Previous research has explored the use of ESWT in individual hand conditions, most notably carpal tunnel syndrome (CTS), however, there is a paucity of agglomerated work on ESWT usage in a spectrum of hand conditions. Current practice in ESWT usage remains largely unregulated, it is heavily dependent on operator preference rather than evidence-based medicine, and there is a notable lack of standardization in delivery protocols. This variability in practice may lead to inconsistencies in treatment outcomes and hinder the ability to compare results across studies. Furthermore, much of the existing literature is restricted to short follow-up periods of less than three months, which are insufficient to determine the durability or true clinical value of ESWT. Identifying and understanding the existing evidence of ESWT usage in hand surgery would enable us to recognize gaps or limitations in the literature and provide valuable insights to design future clinical trials, allowing for more targeted and effective research efforts in this area. This systematic review aims to provide an overview of application of ESWT in hand surgery.

## Methods

### Search strategy

In accordance with the Preferred Reporting Items for Systematic Review and Meta-Analyses (PRISMA) statement,^14^^,^^15^ a search was conducted across Medline and Embase databases for studies that fulfilled inclusion criteria. In addition, the references of included articles and previous meta-analysis were also screened manually for a comprehensive search. The search term used was a permutation of ‘shockwave’ and hand surgery conditions. The details of search strategy can be found in Supplementary Material 1.

*Inclusion Criteria*

Full text English articles including case series, retrospective and prospective cohort studies, cross-sectional studies, and randomized controlled trials which investigated the application of shockwave therapy in hand surgery conditions with at least three months follow up

*Exclusion criteria*

1. Case reports, reviews, commentaries, letter to editors, and editorials
2. Non-English language studies
3. Conference abstract without full text

### Outcome measures

The evaluated outcomes could be categorized into four dimensions: clinical assessment, Patient-Reported Outcome Measures (PROMs), neurophysiological tests, and a miscellaneous category encompassing imaging, patient satisfaction, and Immunohistochemical staining.

### Study selection and data management

Preliminary abstract screening was carried out by two authors (ZYW and OA) independently while a senior independent author (RF) resolved any disputes through consensus. Two independent authors (ZYW and OA) extracted the relevant information from the included articles into a designed proforma. Study characteristics including author, year of study, country of origin, protocol for shockwave therapy, conditions, follow up time, adverse events and assessed outcomes were also extracted. PRISMA flowchart for the selection process and systematic mapping of all included studies based on was constructed. Data were analyzed and narratively summarized using descriptive statistics in tables or graphs for each objective of the study. R Studio (version 4.0.3) was used for visualization of spatial data. Quality assessment of included articles was done with either the JBI critical appraisal tools for case series,^16^ ROBINS-I tools (Risk of Bias in Non-randomized Studies – of Interventions)^17^ or risk-of-bias tools for randomized trials (RoB2)^18^ depending on the study design. Extracted data were used to determine the strength of evidence as per the Grading of Recommendations, Assessment, Development and Evaluation (GRADE) system. The Fragility Index (FI) was calculated using a publicly available free online calculator, using the methods described by Walsh et al.^19^

## Results

### Search and selection

**Figure 1:**
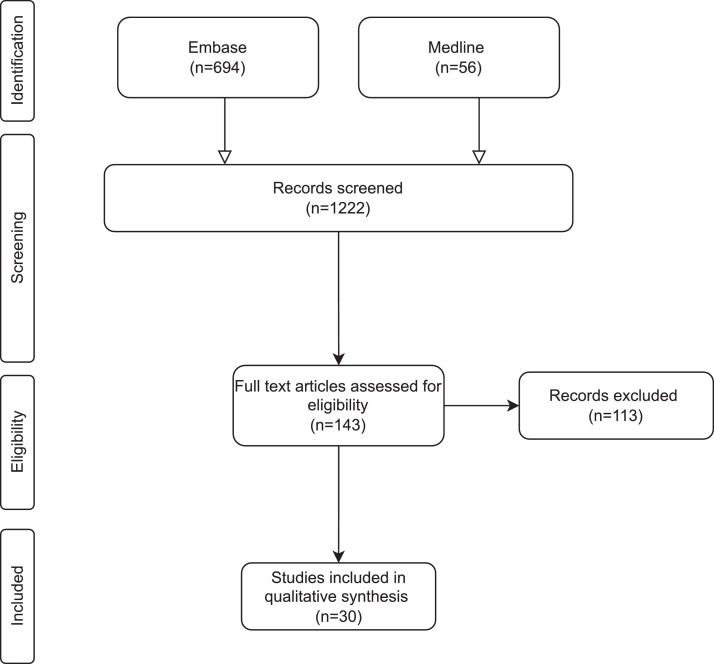
PRISMA flow chart.

The structured searching via search strings returned 1222 studies regarding this topic. Subsequently, 1079 studies were removed during first stage of selection via title and abstract. Further 100 studies were excluded following second stage selection by full text appraisal. Figure 1 summarizes the PRISMA flow diagram for the final 30 reviews for data extraction.

### Characteristics of included studies

**Figure 2:**
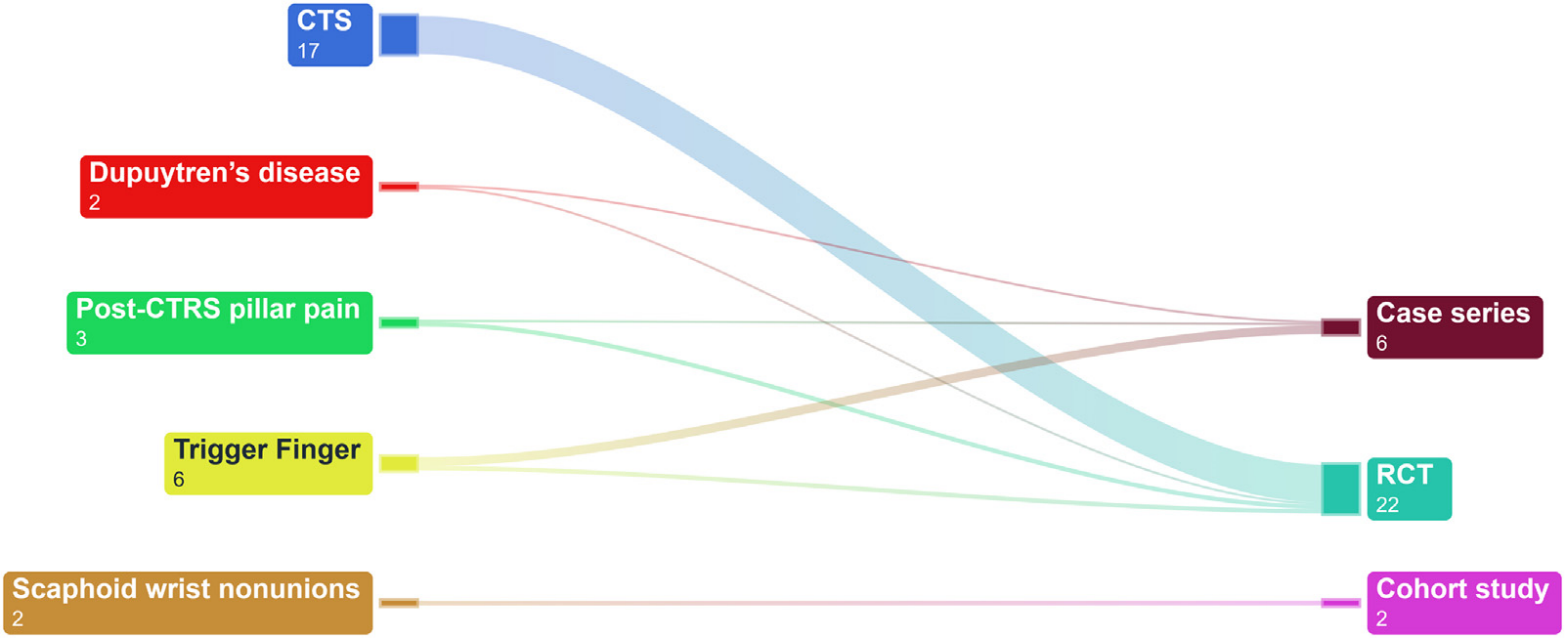
Sankey diagram by study design.

Table 1 provides results of data mapping conducted in this study. This study identified six case series,^20^, ^21^, ^22^, ^23^, ^24^, ^25^ two cohort study^26^^,^^27^ and 22 randomized controlled trials (RCT)^28^, ^29^, ^30^, ^31^, ^32^, ^33^, ^34^, ^35^, ^36^, ^37^, ^38^, ^39^, ^40^, ^41^, ^42^, ^43^, ^44^, ^45^, ^46^, ^47^, ^48^, ^49^, ^50^ (Figure 2). Among the 30 included studies (2143 patients) investigating shockwave therapy in hand surgery conditions, carpal tunnel syndrome was the subject of 17 studies, trigger finger in six, Dupuytren’s disease in two post-CTRS pillar pain in three, and scaphoid wrist nonunions in two. From the 30 reviews, institutions of the main author for the reviews were reported from 12 countries; Turkey (7), Iran (6), Taiwan (4), Italy (3), Austria (3), and one each for Korea, Egypt, Thailand, Germany, China, Poland, and Greece. Most case series were rated as moderate to high quality, whereas the one cohort studies had a moderate risk of bias, and the majority of RCTs showed moderate to high risk of bias (Table 2, Table 3, Table 4). The follow-up time in the included studies ranged from 3 month to 18 months. Eighteen studies had a follow-up of more than 3 months but less than 6 months, while twelve had follow-up durations ranging from 6 months to 1 year. There is variability in shockwave therapy protocol, with the number of sessions ranging from 1 to 18, the total number of shots/pulses ranging from 1000 to 36,000, and energy frequency ranging from 3 to 15, with energy density measured in variable units. About 13% (four out of 30) of studies used shockwave therapy as an adjunct, while 86% (26 out of 30) used it as the primary treatment. Five studies utilized single session ESWT. Eleven studies reported the use of radial extracorporeal shock wave therapy (rESWT), six studies reported the use of focused extracorporeal shock wave therapy (fESWT), and one study used both. The type of shock wave therapy used in the remaining studies was not specified. The most frequently studied clinical assessment was grip and pinch strength, while VAS and BCTQ scores were the most popular patient-reported outcome measures (PROMs) studied. Neurophysiological tests mainly measured sensory nerve conduction velocity (SNCV), distal motor latency (DML), and distal sensory latency (DSL). As shown in Table 5, the overall quality of evidence for the use of shockwave therapy in hand surgery conditions was low to very low, except for carpal tunnel syndrome (CTS), which had high-quality evidence rating. FGI was calculated for all 22 RCTs included, and all trials were found to have an FGI of zero (Table 2, Table 3, Table 4).

**Table 1:** Characteristic of studies.

Year	Author	Study design	Condition(s)	Study details	Comparator group	Total number of sessions	Total number of shots/pulses	Energy frequency (Hz)	Energy density	As primary treatment	As an adjunct treatment	Country	Hospital	Follow up time	No. of patients	Clinical assessment	PROMs	Neurophy-siological test	Others	Adverse events
2011	Romeo. P.	Case series	Post-CTRS pillar pain	ESWT		3	2800	4	0.03 mJ/mm2	Y	N	Milano, Italy	Istituto Ortopedico Galeazzi	4 months	40	Not reported	VAS	Not reported	Not reported	Not reported
2013	Seok, H.	RCT	CTS	ESWT	LCI	1	1000	6	0.09 - 0.29 mJ/mm2	Y	N	Bucheon, Korea	Soonchunhyang University,	3 months	36	Not reported	VAS, Levine Selfassessment Questionnaire	DML, SNAP, CMAP, Semmes-Weinstein testing	Not reported	N
2015	Notarnicola, A.	RCT	CTS	ESWT + median nerve glide exercises + splint	nutraceutical diet + median nerve glide exercises + splint	3	4800	4	0.03 mJ/mm2	Y	N	Bari, Italy	University of Study of Bari	6 months	60	Not reported	VAS, BCTQ, Roles and Maudsley Scores	SNCV, DML	Not reported	Not reported
2015	Paoloni, M.	RCT	CTS	ESWT	US therapy; cryo-US therapy	4	10,000	Not reported	0.05 mJ/mm2	Y	N	Rome, Italy	Sapienza University of Rome,	12 weeks	25	Not reported	VAS, BCTQ	Not reported	Not reported	N
2016	Vahdatpour, B	RCT	CTS	ESWT + splint + NSAIDs	sham ESWT + NSAIDs + Splint + vitamin B1	4	3800	3	0.05, 0.07, 0.1, and 0.15 mJ/mm2	Y	N	Isfahan, Iran	Isfahan University of Medical Sciences	6 months	60	Not reported	VAS, LSQ	DML, SNAP, CMAP	Not reported	Not reported
2016	Ke, M. J.	RCT	CTS	ESWT + splint	single session of ESWT + splint; sham ESWT + splint	3	6000	5	0.003–0.89mJ/mm2	Y	N	Taipei, Taiwan	Tri-Service General Hospital	14 weeks	69	Not reported	BCTQ	CSA, SNCV	Not reported	N
2016	Wu, Y. T.	RCT	CTS	ESWT + splint	sham ESWT + splint	3	6000	5	Not reported	Y	N	Taipei, Taiwan	Tri-Service General Hospital	12 weeks	34	Pinch strength	VAS, BCTQ	CSA, NCV	Not reported	N
2016	Yildirim, P.	RCT	Trigger Finger	ESWT	local corticosteroid injection	3	3000	15	Not reported	Y	N	Bulvari, Turkey	Kocaeli Derince Training and Research Hospital	6 months	40	FT, ST, FIT	NPRS, DASH	Not reported	Not reported	N
2016	Malliaropoulos, N.	Case series	Trigger Finger	ESWT		1	2000	5-6	Not reported	Y	N	Thessaloniki, Greece	Thessaloniki Musculoskeletal Clinic	12 months	44	Not reported	VAS, Roles and Maudsley Scores	Not reported	Not reported	N
2017	Raissi, G. R.	RCT	CTS	ESWT + splint	Splint	3	3000	6	low-energy	N	Y	Arak, Iran	Arak University of Medical Sciences	12 weeks	40	Not reported	NRS, DASH	SNAP, CMAP, DML, DSL	Not reported	Y
2018	Atthakomol, P.	RCT	CTS	ESWT	LCI	1	5000	15	Not reported	Y	N	Chiang Mai, Thailand	Chiang Mai University,	24 weeks	25	Not reported	BCTQ	DML, SNAP, CMAP, DSL	Not reported	Y
2019	Karatas, O.	RCT	CTS	ESWT + splint	sham ESWT + splint	9	13,500	Not reported	0.10 mJ/mm2	Y	N	Ankara, Turkey	Antalya Training and Research Hospital	3 months	49	Grip strength, paresthesia	VAS, MHQ	NCV, SNCV, SNAP, CMAP, DSL	Not reported	N
2019	Haghighat, S.	RCT	Post-CTRS pillar pain	ESWT	sham ESWT	4	2800	4	0.03 mJ/mm2	Y	N	Isfahan, Iran	Isfahan University of Medical Sciences	3 months	40	Not reported	VAS, MHQ	Not reported	Not reported	Not reported
2019	Quadlbauer, S.	Cohort study	Scaphoid wrist nonunions	ESWT + Splint/cast	Splint/cast	1	3000	4	0.41 mJ/mm2	Y	N	Vienna, Austria	AUVA Trauma Hospital Lorenz Böhler - European Hand Trauma and Replantation Center	1 year	42	ROM, Grip strength	VAS, DASH. PRWE, MHQ, MAYO	Not reported	CT scan	Not reported
2020	Xu, D.	RCT	CTS	ESWT + splint + NSAIDs	LCI + splint + NSAIDs	9	9000	6	Not reported	Y	N	Ningbo, China	Medical Treatment Center Lihuili Hospital	12 weeks	55	Not reported	BCTQ	SNAP, CMAP	Not reported	Y
2020	Chang, C. Y.	RCT	CTS	ESWT + PRP	sham ESWT + PRP	1	2000	5	Not reported	N	Y	Taipei, Taiwan	Tri-Service General Hospital	6 months	40	Not reported	BCTQ	CSA, SNCV, DML	Not reported	N
2020	Vahdatpour, B.	Case series	Trigger Finger	ESWT		3	3000	15	Not reported	Y	N	Isfahan, Iran	Isfahan University of Medical Sciences	18 weeks	19	Quinnell grading system	VAS, DASH	Not reported	Not reported	Not reported
2020	Dogru, M.	Case series	Trigger Finger	ESWT		10	20,000	10	Not reported	Y	N	Izmir, Turkey	Dokuz Eylul University	3 months	18	Range of motion, Grip strength, and Pinch strength	NPRS, DASH	Not reported	Not reported	Not reported
2020	Zyluk, A.	Case series	Trigger Finger	ESWT		3	6000	8	Not reported	Y	N	Szczecin, Poland	Pomeranian Medical University	3 months	32	Grade of triggering in Froimson scale	NPRS, Froimson Scale	Not reported	Not reported	N
2021	Ulucakoy, R.	RCT	CTS	ESWT	ESWT+splint; splint; splint+sham ESWT	3	3000	5	0.05 mJ/mm2	Y	N	Ankara, Turkey	Ankara Numune Training and Research Hospital	3 months	189	Not reported	VAS, BCTQ, LANSS	SNCV, DML, DSL	Not reported	N
2021	Elrazik, R. K. A.	RCT	CTS	ESWT	iontophoresis therapy.	18	36,000	Not reported	0.03 mJ/mm2	Y	N	Cairo, Egypt	Modern University for Technology and Information	18 weeks	30	Not reported	VAS	DSL	Not reported	Not reported
2021	Gesslbauer, C.	RCT	CTS	ESWT + splint	sham ESWT + splint	3	1500	4	0.05 mJ/mm2	Y	N	Vienna, Austria	Medical University of Vienna	12 weeks	30	Grip strength	VAS, BCTQ, SF-36	SNCV, DML	Not reported	N
2021	Turgut, M. C.	RCT	Post-CTRS pillar pain	ESWT	sham ESWT	3	6000	5	0.03 mJ/mm2, 400 kPa	Y	N	Trabzon, Turkey	Karadeniz Technical University Medical Faculty,	6 months	60	Pillar pain, Grip strength	VAS, MHQ	Not reported	Not reported	Not reported
2021	Chen, Y. P.	RCT	Trigger Finger	high energy ESWT	low energy ESWT; sham ESWT	4	6000	Not reported	0.01 mJ/mm2, 580 kPa	Y	N	Taipei, Taiwan	Taipei Medical University	6 months	60	FT, ST, FIT	VAS. DASH	Not reported	Not reported	N
2022	Ozturk Durmaz, H.	RCT	CTS	ESWT + splint	LCI + splint ; splint	3	6000	5	Not reported	Y	N	Ankara, Turkey	Ahi Evran University Physical Medicine and Rehabilitation Outpatient Clinic	12 weeks	72	Grip strength	VAS, BCTQ	DML, DSL	Not reported	Y
2022	Saglam, G.	RCT	CTS	ESWT + median nerve glide exercises + splint	Median nerve glide exercises + splint; Median nerve glide exercises + splint + TENS + US	3	6000	5	Not reported	Y	N	Erzurum, Türkiye	Erzurum Regional Training and Research Hospital	3 months	95	Not reported	VAS, BCTQ, LANSS	DML, DSL	Not reported	N
2022	Knobloch, K.	RCT	Dupuytren’s disease	ESWT	sham ESWT	3	6000	Not reported	0.35 mJ/mm2	Y	N	Hannover ,Germany	Hannover Medical School	18 months	52	Grip strength and Dupuytren’s disease progression	VAS, DASH, MHQ, URAM	Not reported	Patient’s satisfaction	N
2022	Taheri, P.	Case series	Dupuytren’s disease	ESWT		6	12,000	3	1.24 mJ/mm2	Y	N	Isfahan, Iran	Isfahan University of Medical Sciences	14 weeks	20	Contracture angle	VAS, DASH	Not reported	Not reported	Y
2023	Gholipour, M.	RCT	CTS	ESWT + LCI	sham ESWT + LCI	4	10,400	4	0.03 mJ/mm2	N	Y	Tehran, Iran	Akhtar Hospital	6 months	40	Not reported	VAS, GSS	NCV	Proportion of participants referred for surgery	N
2023	Quadlbauer, S.	Cohort study	Scaphoid wrist nonunions	two headless compression screws (HCS) vs angular stable scaphoid plate		1	3000	4	0.41 mJ/mm2	N	Y	Vienna, Austria	AUVA Trauma Hospital Lorenz Böhler - European Hand Trauma and Replantation Center	1 year	38	Grip strength, ROM	VAS, DASH. PRWE, MHQ, MAYO	Not reported	CT scan	Not reported

RCT, Randomized controlled trial; CTS, Carpal tunnel syndrome; ESWT, extracorporeal shockwave therapy; PRP, Platelet rich plasma; LCI, Local corticosteroid injection; US, Ultrasound; BCTQ, Boston Carpal Tunnel Questionnaire; VAS, Visual Analog Scale; MHQ, Michigan Hand Outcomes Questionnaire; NRS, Numeric Pain Rating Scale; Quick-DASH, Quick disabilities of the Arm, Shoulder, and Hand Questionnaire; GSS, Global symptom score; ADL, Activities of daily living; LANSS, Leeds Assessment of Neuropathic Symptoms and Signs; LSQ, Levine Self-Assessment Questionnaire; SF-36, 36-item Short Form Health Survey Questionnaire; CSA, Cross-sectional area; SNCV, Sensory conduction velocity; DML, Distal motor latency; NCV, Nerve conduction velocity; SNAP, Sensory nerve action potential; CMAP, Compound motor action potential; DSL, Distal sensory latency.

**Table 2:** Risk of bias of case series using Joanna Briggs Institute (JBI) critical appraisal checklist.

**Table 3:** Risk of Bias using the Risk of Bias for Randomized trials (ROBINS-II) tool.

**Table 4:** Risk of Bias using Risk of Bias in Non-randomized studies – of Interventions (ROBINS-I) tool.

**Table 5:** Assessment of quality using the GRADE system.

Diseases	No. of studies (no. of participants)	Risk of bias	Inconsistency	Indirectness	Imprecision	Publication bias	Factor	Quality
CTS	17 (949)	Serious (-1)	-	-	-	-	Large effect (+1)	High
Trigger Finger	6 (213)	Serious (-1)	-	-	Serious (-1)	Likely (-1)	-	Low
Dupuytren’s disease	2 (72)	-	-	-	Very serious (-2)	Very likely (-2)	-	Very low
Post-CTRS pillar pain	3 (134)	Very serious (-2)	-	-	Very serious (-2)	Likely (-1)	-	Very low
Scaphoid wrist nonunions	2 (80)	-	-	Serious (-1)	Very serious (-2)	Very likely (-2)	-	Very low

### CTS (n = 17,949 patients)

The included studies comprised one study of moderate carpal tunnel syndrome (CTS),^31^ ten studies of mild to moderate CTS^30^^,^^33^, ^34^, ^35^, ^36^^,^^38^^,^^40^^,^^42^, ^43^, ^44^, one study of mild to severe CTS,^28^ and three studies where the severity of CTS was not specified.^29^^,^^32^^,^^39^ Eight studies (546 patients) compared ESWT with sham treatments, physiotherapy, median nerve gliding exercises, or splinting. Seven out of eight studies superior results for ESWT. Particularly there is a trial comparing three-session versus one-session ESWT, which indicated that higher frequency resulted in better outcomes.^33^

All nine studies (403 patients) comparing ESWT with various treatment modalities, including, iontophoresis therapy, local corticosteroid injection (LCI), nutraceutical diet, ultrasound, and cryo-ultrasound therapy, demonstrated clinical benefit. Among these, five studies specifically compared ESWT to LCI. ESWT provided longer-lasting pain relief, with comparable outcomes to LCI at later time points, while LCI offered better short-term pain relief. Similarly, ESWT showed longer-lasting functional improvements, with studies reporting either sustained benefits for ESWT or comparable outcomes after LCI's initial short-term advantage diminished. In contrast, Paoloni et al. demonstrated that ESWT offered superior long-term pain relief at 12 weeks compared to ultrasound or cryo-ultrasound therapy. Chang et al. noted slight improvements in functional scores and nerve conduction when ESWT was combined with platelet-rich plasma (PRP) compared to PRP alone, though the differences were not significant. Notarnicola et al. compared ESWT with a nutraceutical regimen composed of Echinacea, alpha lipoic acid, and conjugated linoleic acid and found comparable improvements in pain and function with no significant differences between the two. Additionally, ESWT was found to be more effective than iontophoresis, as demonstrated by Elrazik et al., showing greater improvements in pain reduction and sensory nerve conduction velocity.

### Trigger finger (n = 6,2132 patients)

The seven included studies (two RCTs, four case series)^21^^,^^22^^,^^24^^,^^48^, ^49^, ^50^ predominantly employed the Quinnell classification and Froimson Scale for categorizing trigger finger, with a primary emphasis on Grade 2 or more severe stages of the condition. In all studies, there was demonstrated superiority in in disease grading, pain, and PROMs. Yildirim et al. conducted a direct comparison, finding that both ESWT and corticosteroid injection led to significant improvements in pain, functional scores, and disease severity over a 6-month period Interestingly, one study demonstrated that high-frequency ESWT resulted in a superior outcome compared to low-frequency ESWT.^50^ The study by Malliaropoulos et al. also found a correlation between symptom duration and the number of sessions required.

### Other hand surgical conditions

Among the four studies included on Dupuytren’s disease (72 patients), although all indicated clinical benefit, only one was an RCT.^20^^,^^45^^,^^51^^,^^52^ Knobloch et al.'s RCT showed significant pain reduction but non-significant changes in PROMs like DASH and MHQ scores and Taheri et al. reporting significant gains in grip strength, DASH scores, and MCP joint angles.

All three studies on CTRS pillar pain demonstrated benefits in pain reduction, with only one study employing PROMs to assess functional outcomes. In the investigation of scaphoid wrist non-union, ESWT was employed as an adjunct for both arms, making it difficult to ascertain superiority and clinical benefit.^26^ In another study by Quadlbauer et al, ESWT as an adjunct therapy resulted in 81% union rates compared to 75% in the non-ESWT group, though the difference was not statistically significant.^27^ However, patients in the ESWT group experienced significantly better pain reduction and Mayo scores, while significant difference improvements in other PROMs were not observed.

### Adverse events

Most studies reported no adverse events, and in those where adverse events were documented, they were typically minor, transient, and self-limiting, often manifesting as temporary pain, erythema, swelling, bruising, tingling, or numbness at the treatment site, all of which resolved without intervention and with no reports of serious complications. While no adverse events were reported in studies utilizing fESWT, 4 out of 11 studies involving rESWT reported adverse events.

## Discussion

Our review on the application of ESWT in hand surgery highlighted that the majority of published research focused on carpal tunnel syndrome (CTS). Most studies reported short-term benefits in clinical and PROMs, with minimal complications. However, there was considerable heterogeneity in shockwave therapy delivery protocols, particularly in intensity and frequency. The administration of shockwave therapy has been suggested to exhibit a dose-dependent effect without major complications, but this was reported in only a few studies. Although numerous studies, particularly those focusing on CTS, have investigated the use of ESWT, uncertainty persists due to the low quality of the available evidence.

The clinical benefit of ESWT in CTS appears to be influenced by disease severity and its comparison with other treatment modalities. Rashad et al. highlighted that ESWT might not be effective in severe CTS, making it the only study specifically evaluating its impact at this stage. While previous studies have demonstrated that ESWT promotes axonal regeneration, potentially accelerating Wallerian degeneration and enhancing axonal recovery, specific data on its efficacy in severe CTS remain lacking.

When compared to conservative management, ESWT showed superiority in-most studies. The lack of demonstrated superiority of ESWT in Ulucaköy et al and Karataş et al, likely stems from methodological limitations and study design factors. Ulucaköy et al showed improvements across all groups, including sham ESWT, suggesting a strong placebo effect or natural symptom resolution, particularly in mild-to-moderate CTS. Additionally, the lack of significant electrophysiological improvements raises questions about the true physiological impact of ESWT in this cohort. Karataş et al, despite using multiple ESWT sessions, failed to show superiority over sham ESWT, potentially due to high variability in baseline characteristics, insufficient standardization of ESWT parameters (intensity, frequency, and number of sessions), and a small sample size, limiting statistical power. These findings suggest that placebo effects, variability in ESWT protocols, short follow-up durations, and effective control interventions likely contributed to the lack of observed superiority in these studies, in contrast to trials with longer follow-ups, optimized treatment protocols, and larger sample sizes, which have generally reported better outcomes with ESWT. Based on the available evidence, ESWT is strongly supported as an effective treatment for mild-to-moderate CTS, either as a standalone therapy or in combination with conservative management, demonstrating superior outcomes.

When compared to other treatment modalities, ESWT was generally superior, except in phonophoresis, which demonstrated comparable outcomes. While phonophoresis may be a potential alternative, the current evidence remains unclear, whereas ESWT has more robust supporting data, reinforcing its role as an effective treatment for CTS. Chang et al. which demonstrated that combination of ESWT didn’t provide additional benefit with PRP used a single ESWT session with a small sample size, which may have contributed to its non-significant findings. Although the GRADE assessment suggested strong evidence, the FGI for all RCTs was zero, indicating that even a minor change in outcomes could nullify the significance of the findings. This contradictory result highlights the need for a large-scale, multinational clinical trial with an adequately powered sample to validate these conclusions.

The range of ESWT energy influx varies, with previous studies categorizing treatment intensity into three levels: low intensity (EFD, <0.08 mJ/mm^2^), medium intensity (EFD, 0.08-0.28 mJ/mm^2^), and high intensity (EFD, >0.28 mJ/mm^2^).^5^ In hand conditions, the majority of studies in this analysis utilized low intensity shockwave therapy. However, three included studies, one focusing on CTS and the other two on trigger finger, investigated the dose dependent effect of ESWT.^33^^,^^48^ All three studies reported a dose-dependent relationship with response, favoring high intensity, as evidenced by significant improvements in patient-reported outcomes and pain reduction. Ke et al. and Chen et al. that higher energy levels resulted in superior symptom relief, functional gains, and longer-lasting effects compared to lower-intensity ESWT. Malliaropoulos et al. further highlighted the importance of treatment frequency, noting that multiple ESWT sessions enhanced outcomes, suggesting that dose dependency may extend beyond energy levels to the number of sessions delivered. Moreover, a study conducted on endothelial progenitor cells of rats in vitro has demonstrated that high-energy shock waves resulted in a decrease in the expression of most cytokines, except for apoptotic factors and fibroblast growth factor 2, ultimately leading to cellular apoptosis.^53^ Nonetheless, the dose-dependent relationship remains associational and is only observed in certain conditions.^54^, ^55^, ^56^ While high intensity energy may yield superior treatment effects with single sessions, it often accompanies increased pain and local swelling, necessitating local anesthesia, which may compromise efficacy. Further studies are needed to evaluate the optimal energy level for ESWT in various clinical settings.

ESWT can be categorized into two types: focused-ESWT and radial-ESWT, which differ in their physical properties and patterns of acoustic wave propagation. Many included studies have not specified which type was utilized, overlooking this distinction, even though comparisons of the two types have been widely explored in other conditions.^57^, ^58^, ^59^, ^60^ Each modality has shown different advantages for various diseases, resulting in inconclusive outcomes. Importantly, no study has directly compared f-ESWT and r-ESWT in hand surgical conditions.

Future directions for ESWT research should prioritize standardizing treatment protocols to enhance patient experience and cost-effectiveness, as current practices vary widely based on physician preference.^61^ One key limitation across the included studies is the lack of follow-up data on subsequent treatments following ESWT. There is little to no mention in most studies regarding whether patients eventually required surgery or alternative interventions, making it difficult to assess the long-term effectiveness of ESWT in preventing disease progression or reducing the need for invasive management. This is particularly relevant for CTS and trigger finger, where some patients may experience only temporary relief with ESWT before progressing to more definitive treatments. Additionally, there is a pressing need for standardization in reporting shockwave parameters to improve reproducibility and interpretation of study findings in musculoskeletal disorders.^62^ Treatment guidelines should differentiate between various forms of therapy, systems, and protocols, rather than treating shockwave therapy as a universal approach.

The application of ESWT in several hand surgical conditions remains understudied, with a notable absence of high-quality research in areas such as flexor tendon injury, de Quervain’s tenosynovitis, Dupuytren’s disease, and hypertrophic hand scars. In flexor tendon injuries, which are typically managed surgically, ESWT may serve as an adjunct to enhance tendon healing, reduce adhesions, and improve tendon gliding. Similarly, its anti-fibrotic and regenerative properties could offer therapeutic benefit in Dupuytren’s disease and hypertrophic scars, while its analgesic and anti-inflammatory effects may be valuable in de Quervain’s tenosynovitis.^63^^,^^64^ Future studies should therefore prioritize well-designed, multicenter randomized controlled trials with adequate power and a minimum of 12 months’ follow-up, incorporating both clinical and patient-reported outcomes. In addition, direct comparisons of radial versus focused ESWT are warranted to clarify their relative safety and efficacy in hand surgery applications, as this distinction remains unexplored in the current literature.

Acknowledging the inherent limitations, this systematic review is faced with several notable challenges. Primarily, the included studies exhibit a considerable risk of bias and present their findings in a heterogeneous manner, thereby hindering the establishment of robust and directly comparable conclusions. Furthermore, the predominant focus on western and developed nations within the reviewed literature restricts the extrapolation of results to low-resource settings. Moreover, the variability in delivery protocols poses a significant obstacle to the feasibility of conducting comprehensive comparative or network meta-analyses. The significance of this review lies in consolidating an otherwise fragmented body of literature and outlining a clear research agenda. Our synthesis demonstrates that, although ESWT shows encouraging short-term benefits in soft tissue conditions of the hand, its definitive role within management pathways remains unresolved. Notably, evidence with follow-up beyond three months is limited to only five conditions: scaphoid wrist nonunions, carpal tunnel syndrome (CTS), Dupuytren’s disease, trigger finger, and post-CTRS pillar pain, leaving its long-term efficacy in other hand pathologies unknown. For ESWT to progress beyond an experimental adjunct and achieve recognition as a validated component of evidence-based hand surgery, future research must address the methodological limitations we have identified.

## Conclusion

Standardization of shockwave therapy frequency and intensity is essential. Although there is an abundance of evidence supporting the use of ESWT in CTS, the overall quality of that evidence remains questionable. Dose-dependent effect remains a possibility but requires further investigation. ESWT demonstrates noteworthy short-term efficacy and is primarily used as an adjunct treatment. Although the literature on CTS is well-developed, meticulously designed RCTs are necessary to further evaluate its efficacy in other hand surgical conditions.

## Funding

No funding was required for this study.

## Ethical statement

The study was conducted in accordance with the Declaration of Helsinki. The study was exempt from IRB review was no confidential patient information was involved.

## Authors’ contributions

All authors approve the final version of the manuscript, including the authorship list and agree to be accountable for all aspects of the work in ensuring that questions related to the accuracy or integrity of any part of the work are appropriately investigated and resolved. All authors have made substantial contributions to all of the following: (1) the conception and design of the study, or acquisition of data, or analysis and interpretation of data, (2) drafting the article or revising it critically for important intellectual content, (3) final approval of the version to be submitted. No writing assistance was obtained in the preparation of the manuscript. The manuscript, including related data, figures and tables has not been previously published and that the manuscript is not under consideration elsewhere.

## Declaration of competing interest

All authors have no conflict of interest.

## Appendix Supplementary materials

Image, application 1
