# Supplementary material for: Systematic mapping review of applications of extracorporeal shockwave therapy (ESWT) in hand surgery
Source: JPRAS Open. 2025 Nov 19;48:356–72. doi: 10.1016/j.jpra.2025.11.017 (PMC12795702; doi:10.1016/j.jpra.2025.11.017)
Supplement: Supplementary file 1 [file mmc1.docx]

Supplementary Table 1 Search strategy

Ovid MEDLINE(R) ALL <1946 to November 08, 2023>

| 1 | shock wave therapy/ | 984 |
| --- | --- | --- |
| 2 | hand/ or hand surgery/ | 48653 |
| 3 | wrist/ | 10910 |
| 4 | finger/ | 32482 |
| 5 | Dupuytren contracture/ | 2938 |
| 6 | tendon/ | 29679 |
| 7 | tenosynovitis/ or trigger finger/ | 3866 |
| 8 | carpal tunnel syndrome/ | 9794 |
| 9 | scaphoid bone/ or carpal bone/ or scaphoid fracture/ | 8053 |
| 10 | 1 and (2 or 3 or 4 or 5 or 6 or 7 or 8 or 9) | 40 |
| 11 | (shock wave or shockwave).ti,ab. | 12281 |
| 12 | (hand or hand surgery or wrist or Finger or Dupuytren* or tendon or tenosynovitis or Trigger finger or Tenosynovitis or Carpal tunnel syndrome or scaphoid bone or carpal bone or scaphoid fracture).ti,ab. | 620632 |
| 13 | 11 and 12 | 526 |
| 14 | 10 or 13 | 528 |

Embase <1974 to 2023 November 08>

| 1 | shock wave therapy/ | 2880 |
| --- | --- | --- |
| 2 | hand/ or hand surgery/ | 40643 |
| 3 | wrist/ | 34854 |
| 4 | finger/ | 31452 |
| 5 | Dupuytren contracture/ | 3432 |
| 6 | tendon/ | 26525 |
| 7 | tenosynovitis/ or trigger finger/ | 7701 |
| 8 | carpal tunnel syndrome/ | 17871 |
| 9 | scaphoid bone/ or carpal bone/ or scaphoid fracture/ | 9169 |
| 10 | 1 and (2 or 3 or 4 or 5 or 6 or 7 or 8 or 9) | 142 |
| 11 | (shock wave or shockwave).ti,ab. | 16321 |
| 12 | (hand or hand surgery or wrist or Finger or Dupuytren* or tendon or tenosynovitis or Trigger finger or Tenosynovitis or Carpal tunnel syndrome or scaphoid bone or carpal bone or scaphoid fracture).ti,ab. | 804039 |
| 13 | 11 and 12 | 650 |
| 14 | 10 or 13 | 694 |
